# Assumptions made when preparing drug exposure data for analysis have an impact on results: An unreported step in pharmacoepidemiology studies

**DOI:** 10.1002/pds.4440

**Published:** 2018-04-17

**Authors:** Stephen R. Pye, Thérèse Sheppard, Rebecca M. Joseph, Mark Lunt, Nadyne Girard, Jennifer S. Haas, David W. Bates, David L. Buckeridge, Tjeerd P. van Staa, Robyn Tamblyn, William G. Dixon

**Affiliations:** ^1^ Arthritis Research UK Centre for Epidemiology, Centre for Musculoskeletal Research, School of Biological Sciences, Manchester Academic Health Science Centre The University of Manchester Manchester UK; ^2^ Department of Epidemiology, Biostatistics and Occupational Health McGill University Montreal Quebec Canada; ^3^ Clinical and Health Informatics Research Group McGill University Montreal Quebec Canada; ^4^ Brigham and Women's Hospital Boston MA USA; ^5^ Health eResearch Centre, Farr Institute for Health Informatics Research The University of Manchester Manchester UK; ^6^ Faculty of Science, Division of Pharmacoepidemiology and Clinical Pharmacology Utrecht University Utrecht The Netherlands; ^7^ Department of Medicine McGill University Montreal Quebec Canada; ^8^ NIHR Manchester Biomedical Research Centre Manchester University NHS Foundation Trust, Manchester Academic Health Science Centre Manchester UK; ^9^ Rheumatology Department Salford Royal NHS Foundation Trust Salford UK

**Keywords:** data preparation, pharmacoepidemiology, reproducibility, transparency

## Abstract

**Purpose:**

Real‐world data for observational research commonly require formatting and cleaning prior to analysis. Data preparation steps are rarely reported adequately and are likely to vary between research groups. Variation in methodology could potentially affect study outcomes. This study aimed to develop a framework to define and document drug data preparation and to examine the impact of different assumptions on results.

**Methods:**

An algorithm for processing prescription data was developed and tested using data from the Clinical Practice Research Datalink (CPRD). The impact of varying assumptions was examined by estimating the association between 2 exemplar medications (oral hypoglycaemic drugs and glucocorticoids) and cardiovascular events after preparing multiple datasets derived from the same source prescription data. Each dataset was analysed using Cox proportional hazards modelling.

**Results:**

The algorithm included 10 decision nodes and 54 possible unique assumptions. Over 11 000 possible pathways through the algorithm were identified. In both exemplar studies, similar hazard ratios and standard errors were found for the majority of pathways; however, certain assumptions had a greater influence on results. For example, in the hypoglycaemic analysis, choosing a different variable to define prescription end date altered the hazard ratios (95% confidence intervals) from 1.77 (1.56‐2.00) to 2.83 (1.59‐5.04).

**Conclusions:**

The framework offers a transparent and efficient way to perform and report drug data preparation steps. Assumptions made during data preparation can impact the results of analyses. Improving transparency regarding drug data preparation would increase the repeatability, reproducibility, and comparability of published results.

**KEY POINTS**

- We have developed an algorithm to prepare raw prescription data for analysis which allows users to select from multiple decisions and assumptions.
- We demonstrate that decisions made during drug data preparation can influence subsequent results.
- The algorithm and framework allow efficient and transparent performance and reporting of data preparation steps.
- Future work will expand the algorithm to be usable across multiple research databases and to include additional features such as handling dose.

## 1 INTRODUCTION

Anonymised electronic health records (EHR) are increasingly used for observational research, especially pharmacoepidemiology studies.^1^ For drug effectiveness and safety studies, EHR research databases provide data on drug exposure and health outcomes. However, because the primary purpose of the EHR is to support clinical care, the data collected are not necessarily of research quality. Consequently, researchers using such data sources need to prepare the data for scientific analysis.

Data preparation incorporates data cleaning, restructuring data into a format appropriate for statistical analysis, and categorisation of variables. While the Strengthening the Reporting of Observational Studies in Epidemiology guidelines^2^ recommend the reporting of how variables are categorised, they make no recommendations regarding cleaning and formatting. Hence, these 2 steps are rarely described adequately in publications. Furthermore, most modern epidemiological textbooks provide little concrete guidance or discussion about this issue.^3^

The more recent Reporting of studies Conducted using Observational Routinely‐collected health Data (RECORD) Statement recommends the sharing of data cleaning methods and any data preparation algorithms.^4^ However, no established framework exists for reporting the steps taken in preparing drug exposure data from EHR. Poor reporting and little sharing of methods between groups mean the methods for preparing medication data remain opaque and inefficient. Appropriate documentation of data preparation procedures would increase transparency, and thus, improve the repeatability and reproducibility, of published results.^5^, ^6^, ^7^

It is recognised that the definition of drug exposure, for example, using prescribed duration vs fixed time windows,^8^ can impact study outcomes. However, we are unaware of any studies to date that explicitly examine the impact of the many small choices made during cleaning and preparation of drug data on study outcomes. Evidence of an impact would strengthen the argument for increased transparency in reporting data preparation steps.

The aims of this study were (1) to develop a framework to define and document decision nodes when preparing raw prescription data recorded in a UK EHR research database and (2) to examine the impact of changing assumptions made at the various decision nodes, using the clinical example of cardiovascular events (CVE) following exposure to oral hypoglycaemics in patients with diabetes. As decisions made during drug data preparation are likely to have different effects on different patterns of drug use, an additional clinical example of CVE following exposure to oral glucocorticoids in patients with rheumatoid arthritis was also examined as oral glucocorticoids represent a more intermittent therapy than long‐term oral hypoglycaemics.

## 2 METHODS

### 2.1 Setting

The setting was the Clinical Practice Research Datalink (CPRD), a UK database of anonymised primary care EHRs covering an active population of around 4.4 million people. Only adult patients (18 years and over) were included. The study was approved by the CPRD ISAC Committee (ISAC protocol 11_154A).

### 2.2 Example 1: Oral hypoglycaemics and CVE in patients with type 2 diabetes

In this example, the study window was from January 1, 2009 to February 29, 2012. Patients with type 2 diabetes were identified from CPRD (see Data S1 for details). Patients were included if their first prescription for an oral hypoglycaemic drug occurred during the study window. Patients with prescriptions for oral hypoglycaemics prior to January 1, 2009 (prevalent users) were excluded from the analysis. Individual patients were followed from the date of their first prescription for oral hypoglycaemics during the study window until transfer out of GP practice, GP practice last collection date, death, or February 29, 2012. Patients with gestational diabetes and polycystic ovaries (an alternative indication for metformin) were excluded (codelists for all variables available in Data S2).

Oral hypoglycaemic drugs were categorised into biguanides, sulphonylureas, and other oral hypoglycaemics. Patients who used insulin during the course of the follow‐up were excluded from the analysis.

### 2.3 Example 2: Oral glucocorticoids and CVE in patients with rheumatoid arthritis

For this example, 2 analyses were performed, the first with a 3‐year study window (January 1, 2008 to October 1, 2011) and the second with a 20‐year study window (January 1, 1992 to October 1, 2011). Patients with incident rheumatoid arthritis (RA) were identified from the CPRD based on an algorithm designed by Thomas et al^9^ (details in Data S1). Patients were included if they were diagnosed with RA during the study window and had at least one prescription for an oral glucocorticoid drug after their RA diagnosis. Individual patients were followed‐up from the date of their RA diagnosis to transfer out of GP practice, GP practice last collection, death, or October 1, 2011. Prevalent RA cases were excluded from the analysis.

### 2.4 Algorithm definition

The aim of the algorithm was to transform raw prescription data into a matrix of drug use through time ready for analysis. For this initial study, dosage was agreed to be out of scope. The planned output was therefore a binary variable (currently exposed or unexposed to the medication), with exposure status varying over time according to the start and end of prescriptions.

Raw prescription data for the example medications were extracted from CPRD using information from the “therapy” and “product” files. For each prescription record, the information available includes a (product) code identifying the medication prescribed, the total quantity prescribed (“qty”), the date a prescription was written, and the duration of a prescription (“numdays”). In addition, the following variables are derived by CPRD from the free text written by GPs for individual prescriptions: “dose_duration,” an estimated prescription duration available for approximately 1% of records, and the numeric daily dose (“ndd”), available for 95% of prescriptions. The date a prescription stopped is not provided and therefore needs to be calculated from one of the measures of duration. It is possible to have multiple prescriptions for the same product code on the same day and prescriptions that overlap.

Two independent groups (RT/NG/DBu and WD/TS/ML/SP) identified the steps required to transform raw prescription data. Discussion between the groups generated a list of decision nodes with assumption options at each node. The list was reviewed by a third group (JH/DBa) who suggested additional decisions, assumptions, or modifications. A final list was agreed by the whole group. Disagreement was resolved through discussion.

**Figure 1:**
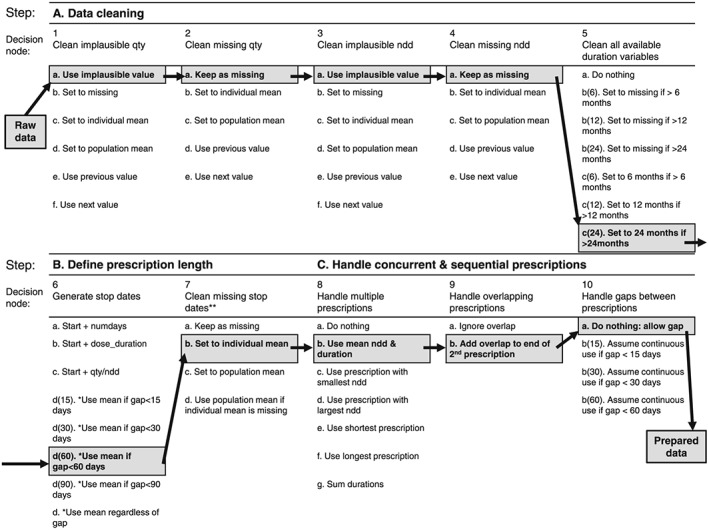
The drug exposure preparation algorithm. qty = total quantity entered by GP for the prescribed product; ndd = derived numeric daily dose; numdays = number of treatment days; dose_duration = derived duration of prescription. The highlighted pathway is the “primary preparation pathway” we defined in the second phase of each analysis; this pathway was used to generate one dataset, then further datasets were generated by varying a single assumption with respect to this primary pathway. All options that produce a missing value stay coded as missing unless otherwise stated. *For options 6d: If only one stop available, use it; if 2 available and equal, use that date; if 2 available and unequal (but within x days), use mean; if 3 available and unequal, use mean of closest 2 if within x days. **Records with missing stop dates after step 7 are dropped

The agreed decision nodes and assumption options (Figure 1) included 10 decision nodes, incorporating 54 plausible assumption options. Data S3 has a detailed description of each decision. The translation of raw data into a drug matrix took place in 3 broad steps. Step A (data cleaning) sought to correct values of number of tablets per prescription, number of tablets per day, missing data, and clinically improbable prescription durations. Cut‐offs for clinical plausibility were defined for each individual product code using the British National Formulary guidelines and clinical experience. Step B aimed to define individual prescription lengths by selecting a stop date from a range of options, or rules to handle missing stop dates. Step C provided options for how to deal with overlapping prescriptions and potentially continuous sequential prescriptions with short gaps between prescriptions.

### 2.5 Cardiovascular events

Cardiovascular events were identified from the events file using Read codes representing acute myocardial infarction, acute coronary syndrome, or stroke (provided in Data S2). Cardiovascular events were defined as the first date in which an event occurred within the follow‐up time.

### 2.6 Covariates

The purpose of the analysis was to examine the impact of assumptions made during data preparation on subsequent results, rather than to examine the safety of oral hypoglycaemics or glucocorticoids. Therefore, no covariates were included.

### 2.7 Analysis

Descriptive statistics were used to characterise the study cohorts. Both oral hypoglycaemic and glucocorticoid drug use were determined as being on or off therapy at the time of the event. The influence of oral hypoglycaemic drug class on the risk of CVE was examined using Cox proportional hazards modelling, with subjects on biguanides as the referent group. The results for “other” oral hypoglycaemics are not reported for simplicity. The influence of oral glucocorticoids on risk of CVE was also examined using Cox modelling, with subjects off therapy as the referent group. The results are expressed as hazard ratios (HR) and 95% confidence intervals (CI). A two‐stage approach was taken to examine variability in results obtained from different data preparation pathways. Firstly, 50 random data preparation pathways were run for each of the 3 analyses. Cox modelling was conducted on each dataset, and the distributions of HRs and SEs were examined graphically using boxplots. Secondly, the group defined a primary data preparation route by listing their preferred combination of assumptions (highlighted in Figure 1). The primary data preparation route was executed first then secondary datasets were created by changing one option in one decision node. Secondary datasets were created for all possible assumption options at each node, keeping the primary pathway fixed at all but one decision node. The analysis was conducted using Stata version 12.1 (http://www.stata.com).

## 3 RESULTS

### 3.1 Oral hypoglycaemics and CVE in patients with type 2 diabetes

Between January 1, 2009 and February 29, 2012, a total of 38 902 patients were identified with 719 344 prescriptions for oral hypoglycaemic medication and 4162 CVEs during the study window. In the raw prescription data, the distribution of missing data was as follows: 12.5% missing numeric daily dose (ndd), 97.2% missing numdays, and 99.9% missing dose_duration.

**Figure 2:**
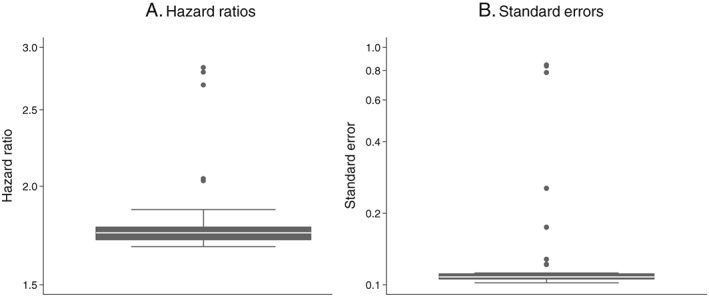
Influence of drug exposure data preparation assumptions on association between oral hypoglycaemic drug class (sulfonylureas compared with biguanides as referent) and CVD events: Distribution of hazard ratios and standard errors from 50 random data preparation pathways

The distributions of HRs and SEs obtained from analysing 50 random data preparation pathways are shown in Figure 2A,B. Very similar HRs and SEs were observed for the majority of the pathways. The median HR was 1.74 and the median SE was 0.11. Six pathways produced outlying HRs of greater than 2.0 and outlying SEs of greater than 0.17, all of which used either option 6a (calculating the stop date from start date + “numdays”) or 6b (calculating the stop date from start date + “dose_duration”). Point estimates could not be calculated for 3 pathways, all using option 6b, due to a high proportion of missing data.

**Figure 3:**
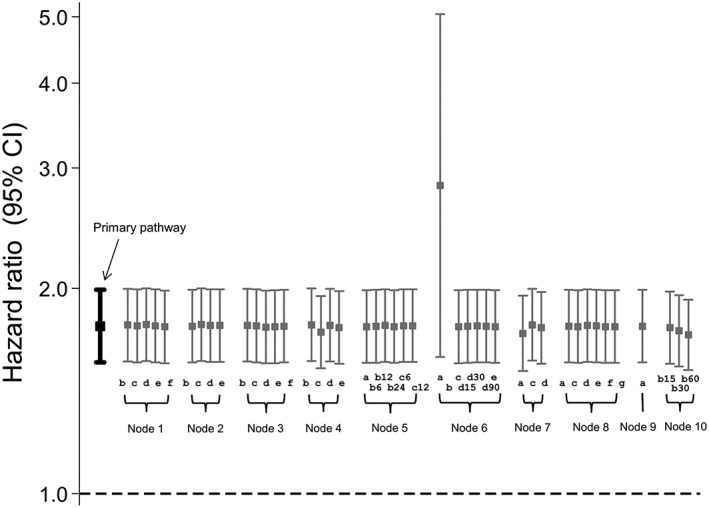
Influence of drug exposure data preparation assumptions on association between oral hypoglycaemic drug class (sulfonylureas compared with biguanides as referent) and CVD events: Effect of changing one data preparation option from primary pathway

Figure 3 shows the results obtained using the primary data preparation pathway and all possible secondary pathways. For the primary pathway, patients on sulphonylureas were 77% more likely to experience a CVE compared with those on biguanides (HR = 1.77; CI, 1.56‐2.00). Changing the data preparation options produced very similar results for the majority of decisions, with HRs ranging from 1.75 to 1.77. Option 4c (set missing “ndd” to the population mean “ndd” for that product code) produced a slightly lower HR than the primary preparation pathway, with a 72% increased risk of CVE amongst sulphonylurea users (HR = 1.72; CI = 1.53, 1.95). Option 6a resulted in an almost threefold increased risk of a CVE for those on sulphonylureas compared with biguanides (HR = 2.83; CI, 1.59‐5.04). An HR could not be calculated after selecting option 6b as the majority of the data were missing for the “dose_duration” variable. Option 7a (keep missing stop dates as missing) and options 10b(30) and 10b(60) of node 10 (assume continuous use if gaps between prescriptions are <30 days or <60 days) produced results that were slightly lower in magnitude than the primary data preparation pathway.

### 3.2 Oral glucocorticoids and CVE in patients with RA: 3‐year follow‐up

Between January 1, 2008 and October 1, 2011, a total of 2377 incident RA patients were identified with 30 493 prescriptions for oral glucocorticoid medication and 103 CVEs during the study window. In the raw prescription data, the distribution of missing data was as follows: 50% missing ndd, 97% missing numdays, and 99.2% missing dose_duration.

**Figure 4:**
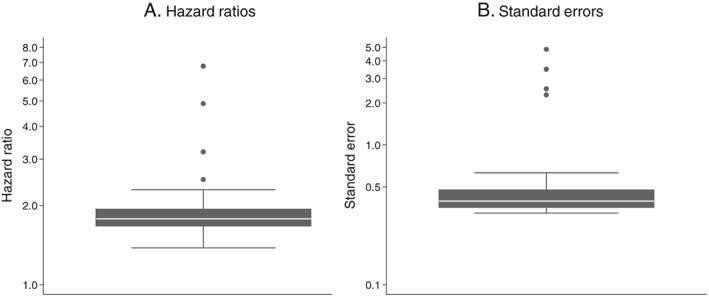
Influence of drug exposure data preparation assumptions on association between oral glucocorticoid use (on vs off) and CVD events: Distribution of hazard ratios and standard errors from 50 random data preparation pathways; 3 years of follow‐up

As for oral hypoglycaemics, similar HRs and SEs were observed for the majority of the 50 random data preparation pathways (Figure 4). The median HR was 1.78, and the median SE was 0.40. Four pathways, all using either option 6a or 6b, produced outlying HRs of greater than 2.5 and outlying SEs of greater than 2.0. For one pathway, using option 6b, a point estimate could not be calculated.

**Figure 5:**
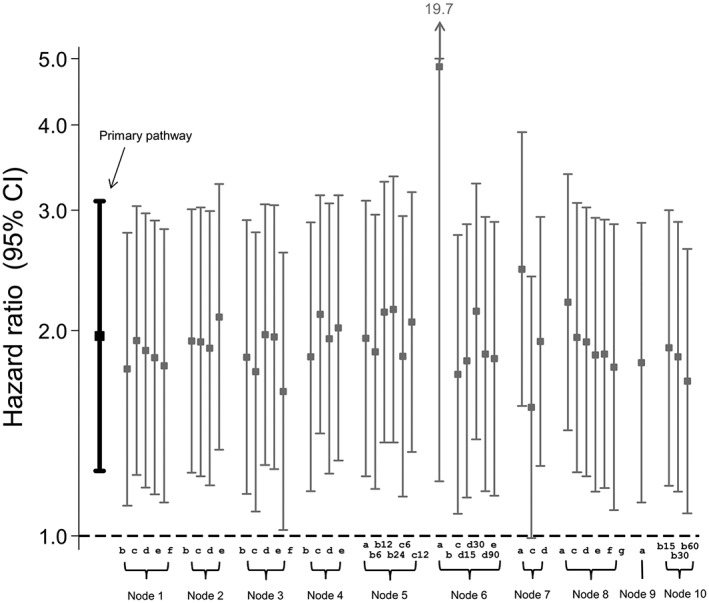
Influence of drug exposure data preparation assumptions on association between oral glucocorticoid use (on vs off) and CVD events: Effect of changing one data preparation option from primary pathway; 3 years of follow‐up

For the primary data preparation pathway, current glucocorticoid users were almost twice as likely as non‐users to experience a CVE (HR = 1.96; CI, 1.25‐3.09) (Figure 5). The majority of the secondary pathways produced broadly similar results, with HRs ranging from 1.63 to 2.28. As before, option 6a produced a markedly higher HR (HR = 4.86; CI, 1.20‐19.67) and an HR could not be calculated for option 6b. Option 7a produced a slightly higher HR (HR = 2.46; CI, 1.55‐3.90) and option 7c (set missing stop date to mean for the population) produced a slightly lower HR with confidence intervals that spanned one (HR = 1.54; CI, 0.99‐2.40).

### 3.3 Oral glucocorticoids and CVE in patients with RA: 20‐year follow‐up

Between January 1, 1992 and October 1, 2011, a total of 12 786 incident RA patients were identified with 369 738 prescriptions for oral glucocorticoid medication and 2565 CVEs during the study window. In the raw prescription data, the distribution of missing data was as follows: 39.8% missing ndd, 93.4% missing numdays, and 99.5% missing dose_duration.

**Figure 6:**
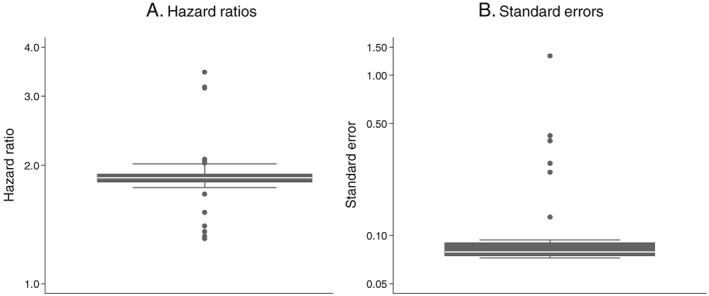
Influence of drug exposure data preparation assumptions on association between oral glucocorticoid use (on vs off) and CVD events: Distribution of hazard ratios and standard errors from 50 random data preparation pathways; 20 years of follow‐up

Very similar HRs and SEs were observed for the majority of the 50 random data preparation pathways (Figure 6), although slightly more variability was observed in this setting, particularly with respect to HRs. The median HR was 1.86, and the median SE was 0.08. Three outlying HRs of greater than 3.0 were observed and 6 outlying HRs of less than 1.7 were observed. Five pathways generated outlying SEs of greater than 0.13, with one producing a very large SE of 1.3. All pathways producing an outlying HR or SE used either option 6a or 6b.

**Figure 7:**
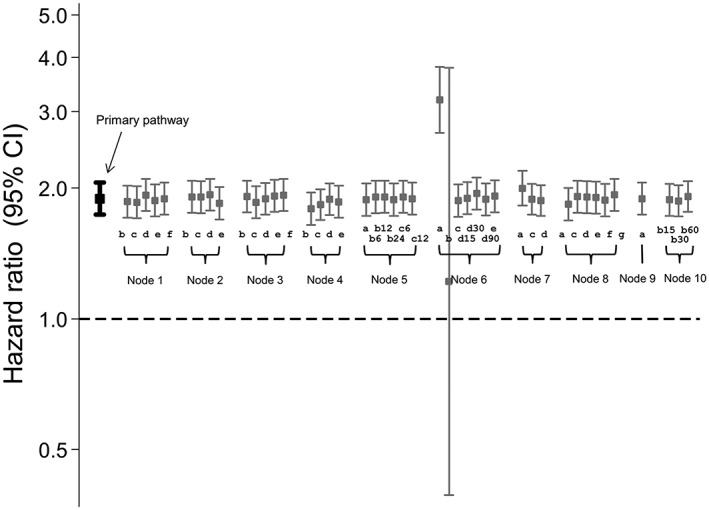
Influence of drug exposure data preparation assumptions on association between oral glucocorticoid use (on vs off) and CVD events: Effect of changing one data preparation option from primary pathway; 20 years of follow‐up

For the primary data preparation pathway, compared with patients not taking oral glucocorticoids, those who did were almost twice as likely to have a CVE (HR = 1.89; CI, 1.74‐2.06) (Figure 7). The majority of secondary pathways produced very similar results, with HRs ranging from 1.79 to 1.94. However, option 6a generated a higher HR (HR = 3.19; CI, 2.68‐3.80) and option 6b produced a lower HR with wide confidence intervals that spanned one (HR = 1.22; CI, 0.39‐3.78). In addition, option 7a generated a slightly higher HR (HR = 2.00; CI, 1.82‐2.19).

## 4 DISCUSSION

We developed a framework for transparently preparing raw prescription data from an EHR research database for analysis, in the form of a generic algorithm, and applied this framework to data from the CPRD. It has previously been demonstrated that high‐level decisions about drug exposure definition, for instance, whether a fixed time window or the prescribed duration is used,^8^, ^10^ can influence study results. In this study, we demonstrate the influence of much smaller‐scale decisions (ie, steps taken to define “prescribed duration”) on study results. This highlights the importance of full transparency in study methodology for replication of results.

While the results were similar for the majority of the pathways tested, certain assumptions appear to have a greater influence on results. In particular, options 6a and 6b led to markedly different HRs compared with the primary path (Figures 3, 5, and 7). Decision 6 is the step in which a stop date is assigned to each prescription and options 6a and 6b assign one of the 2 duration variables provided in the raw data (numdays and dose_duration); this would therefore be a reasonable approach to take. However, both of these variables have a high proportion of missing values which could result in many incomplete records being dropped in later decisions: This is likely the reason for the atypical results produced.

We observed more variation in the 3‐year oral glucocorticoid results, representing intermittent prescribing, compared with the oral hypoglycaemic results, representing continuous prescribing. In the 20 year oral glucocorticoid results, we observed more variation when testing the 50 random pathways but less variation when comparing secondary pathways to the primary pathway. This perhaps indicates that the impact of data preparation decisions may differ according to the length of follow‐up.

There are some limitations to consider when interpreting these results. We did not examine all possible data preparation pathways as there were in excess of 11 000 and each preparation pathway required considerable computing time. It is possible that certain combinations of assumptions could have had a larger impact on the results and our findings are an underestimate of the full range of variability in the results. Changing the order in which the decision nodes are executed may also impact the results. The algorithm was developed using CPRD data from the UK and may not be immediately transferrable when preparing drug data from other EHR research databases, particularly non‐UK databases. Furthermore, the impact of data preparation decisions might be different in different databases and countries.

The framework presented in this paper represents a first attempt to formalise the steps taken to prepare drug exposure data from EHR which we plan to expand on in future work. The current algorithm does not explicitly include assumptions for calculating drug dose. Additional assumptions, within the current nodes or at new decision nodes, are also possible. Planned refinements include allowing a choice of average measurement (ie, median and mode as well as mean), processing at the drug‐substance level rather than specific product code level, and incorporating a researcher‐specified order of preference when selecting duration. Furthermore, we aim to develop the algorithm so it can be used on non‐CPRD EHR data. The algorithm and Stata code are available for download on http://Zenodo.org 11 and future updates will also be made publicly available. According to a New York Times article, data scientists estimate spending 50% to 80% of their time preparing datasets for analysis,^12^ meaning a shared algorithm might significantly increase the efficiency of many research groups. The goal of increasing transparency, by using the algorithm as a framework for reporting steps in data preparation, aligns with recently published recommendations from the RECORD initiative.^4^

This study has focussed on the importance of transparency for replicability. It has purposefully not presented a recommended route for data preparation, and while a “primary pathway” was defined for the purposes of the paper this is not to be taken as a “correct” path. Appropriate data preparation will depend on many factors including the drug type and typical prescribing patterns. Ideally, individual studies should attempt to validate their exposure definitions or to quantify the risk of misclassification bias. The algorithm enables the efficient preparation of multiple datasets, as in this study, and testing multiple datasets would indicate how typical the output of a particular pathway was. However, this would not imply the result was unbiased.

In conclusion, we have developed a drug preparation algorithm for calculating drug exposure from raw prescription data and have shown that there are a large number of potential data preparation pathways. The majority of assumptions made when preparing drug exposure data for analysis in pharmacoepidemiological studies did not influence the results in these 2 exemplar studies, but assumptions involving the calculation of stop dates did have a substantial impact, meaning it is vital to be transparent about assumptions made in data preparation. More explicit and transparent reporting of how drug data are prepared for analysis is therefore crucial and would increase the repeatability, reproducibility, and comparability of published results.

## ETHICS STATEMENT

The study was approved by the CPRD Independent Scientific Advisory Committee (ISAC protocol 11 154A).

## PRIOR POSTINGS STATEMENT

This manuscript contains original unpublished work and has not been submitted for publication elsewhere. Parts of the work have been presented at the following conferences: 29th International Conference on Pharmacoepidemiology & Therapeutic Risk Management, August 2013 (abstract 748, Movahedi et al.); and Informatics for Health, April 2017 (Joseph R et al.).

## Supporting information

Data S1. Definitions of diagnoses and medication use.

Data S2. Supporting information

Data S3. Expanded description of drug data preparation algorithm
