# Supplementary material for: Assumptions made when preparing drug exposure data for analysis have an impact on results: An unreported step in pharmacoepidemiology studies
Source: Pharmacoepidemiol Drug Saf. 2018 Apr 17;27(7):781–8. doi: 10.1002/pds.4440 (PMC6055712; doi:10.1002/pds.4440)
Supplement: Supplementary file 1 — Data S1. Definitions of diagnoses and medication use. [file PDS-27-781-s001.pdf]

Supplementary File 1 – definitions of diagnoses and medication use

Type 2 Diabetes (inclusion criterion)

Definition: at least one record with a Read code for type 2 diabetes. Disease onset taken to be the date of the first recorded Read code.

Rheumatoid arthritis (inclusion criterion)

Definition: patients with rheumatoid arthritis (RA) were defined using an algorithm developed by Thomas et al (*Arthritis Rheum 2008; 59(9):1314-21. doi:10.1002/art.24015*). The algorithm is summarised in the figure below. Onset of RA was taken to be the date of the first RA Read code or first disease-modifying anti-rheumatic drug (DMARD) prescription.

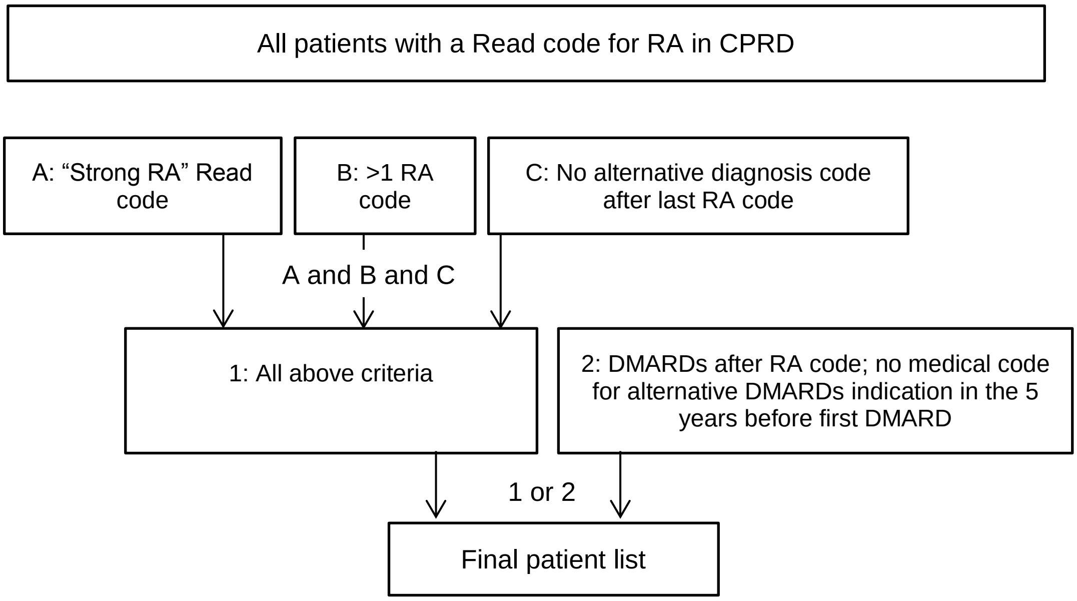

Gestational diabetes (exclusion criterion)

Definition: at least one record with a Read code for gestational diabetes. Patients were excluded if they ever had a Read code for gestational diagnosis.

Polycystic ovaries (exclusion criterion)

Definition: at least one record with a Read code for polycystic ovaries. Patients were excluded if they ever had a Read code for polycystic ovaries.

Insulin (exclusion criterion)

Definition: patients with any prescription with one of the following codes during follow-up were excluded

Oral hypoglycaemic medication (exposure)

Definition: any prescription with one of the listed product codes

Oral glucocorticoids (exposure)

Definition: any prescription with one of the listed product codes

Cardiovascular Events (outcome)

Definition: at least one record with a Read code for the listed cardiovascular events. Disease onset taken to be the date of the first recorded Read code. Cardiovascular events were myocardial infarction, stroke, and ischaemic heart disease.
