# Supplementary material for: Assumptions made when preparing drug exposure data for analysis have an impact on results: An unreported step in pharmacoepidemiology studies
Source: Pharmacoepidemiol Drug Saf. 2018 Apr 17;27(7):781–8. doi: 10.1002/pds.4440 (PMC6055712; doi:10.1002/pds.4440)
Supplement: Supplementary file 3 — Data S3. Expanded description of drug data preparation algorithm [file PDS-27-781-s003.pdf]

Supplementary File 3 – Expanded description of drug data preparation algorithm

Overview

In CPRD, individual prescriptions have a date of issue but no end date. This algorithm determines the start and stop date of individual prescriptions. Initially, the issue date is taken to be the start date. An end date is then determined by estimating the duration of each prescription based on multiple sources of information within the raw CPRD data. The algorithm takes into account the following sources of duration information:

- The prescription duration as entered by the prescriber (numdays)
- A duration variable derived from free text notes written by the prescriber (dose_duration)
- The quantity (qty) of drug units (e.g. tablets) in a prescription divided by the daily dose (ndd). The daily dose variable is the number of drug units to be taken per day and is also derived from the free text notes written by the prescriber.

Once each prescription is assigned a start and end date, longitudinal exposure history is assessed. This assessment is made at the product level. Consecutive prescriptions may overlap. The algorithm includes an option to move the start dates of consecutive prescriptions so they no longer overlap, assuming that the patient completes one course before beginning the next. Finally, there may be small gaps between consecutive prescriptions. It is possible that these gaps are artefacts resulting from previous data cleaning steps rather than true breaks in drug exposure. The algorithm therefore includes options to “fill in” these gaps, reclassifying them as exposed time.

Explanation of each decision node

Decisions 1 through 4 perform data cleaning for the variables qty and ndd, which are used to generate one of the duration variables used in Decision 5.

- *Decision 1) Handle implausible qty* - “Plausible” values need to be pre-specified (e.g. based on prescribing guidelines and clinical experience). Values outside this range may be [1a] ignored, [1b] set to missing, or imputed. Imputation options include: [1c] set to the mean value for that patient for that product code, [1d] set to the mean value for the whole cohort for that product code, [1e] carry forward the previous value for that patient for that product code, or [1f] carry backward the next value for that patient for that product code.
- *Decision 2) Handle missing qty* – Options for missing qty are: [2a] leave as missing, [2b] set to the mean value for that patient for that product code, [2c] set to the mean value for the whole cohort for that product code, [2d] carry forward the previous value for that patient for that product code, or [2e] carry backward the next value for that patient for that product code.
- *Decision 3) Handle implausible ndd* – “Plausible” values need to be pre-specified (e.g. based on prescribing guidelines and clinical experience). Options for implausible ndd are the same as for decision 1.
- *Decision 4) Handle missing ndd* – Options for missing ndd are the same as for decision 2.

Decision 5 cleans implausibly high values for each of the three available duration variables (numdays, dose_duration, and qty/ndd).

- *Decision 5* – options for cleaning each duration variable are: [5a] make no changes, [5b(X)] set to missing if duration is greater than X months, or [5c(X)] set to X if duration is greater than X months. X is 6, 12, or 24.

Decision 6 defines a stop date for each prescription, either by choosing one of the available duration variables or by taking an average of them. **Decision 7** then handles any prescriptions that have not been assigned a stop date. Prescriptions with missing stop date after Decision 7 are dropped.

- *Decision 6* – calculate stop date as prescription start date + one of the following duration definitions: [6a] numdays, [6b] dose_duration, [6c] qty/ndd, or [6d(X)] where only one variable is available, use that variable; if at least two variables are available and equal, use the matching value; otherwise, take the mean of the two nearest values. If the difference between the two nearest values is greater than X, set to missing. (X is 15, 30, 60, or 90 days, or no upper limit).
- *Decision 7* – if stop date is missing: [7a] keep as missing, [7b] set to the mean value for that product code for that patient, [7c] set to the mean value for that product code for the whole cohort, [7d] set to the mean value for that product code for that patient, otherwise set to the mean value for that product code for the whole cohort.

Decisions 8 and 9 handle prescriptions for which the start and stop dates overlap. First, decision 8 deals with multiple prescriptions issued on the same date, and then decision 9 handles any remaining overlaps.

- *Decision 8* – for multiple prescriptions for the same product code on the same day, but with different stop dates, options are: [8a] do nothing, overlaps roll over to decision 9; [8b] calculate the mean duration of prescriptions and drop redundant records; [8c] keep the record with the smallest ndd; [8d] keep the record with the largest ndd; [8e] keep the record with the shortest duration; [8f] keep the record with the longest duration; [8g] sum the durations and drop redundant records.
- *Decision 9* – for consecutive records with overlapping start and stop dates, options are [9a] to ignore the overlap - if dose is calculated after running the algorithm, this option will lead to implicitly summed doses; [9b] move the overlapping time to the next treatment gap – this assumes patients finish one course before starting the next.

Decision 10 handles small gaps between consecutive prescriptions by either allowing these gaps to remain classified as unexposed or reclassifying the gaps as exposed when the gap is less than a specified number of days.

- *Decision 10* - [10a] do nothing – the gap remains classified as unexposed; [10b(X)] move the stop date of the preceding prescription to “fill in” the gap, reclassifying the time as exposed, if the gap between consecutive prescriptions is less than X days. X is 15, 30, or 60.
